# Implementing a new EPR lineshape parameter for organic radicals in carbonaceous matter

**DOI:** 10.1186/1751-0473-8-15

**Published:** 2013-07-17

**Authors:** Mathilde Bourbin, Yann Le Du, Laurent Binet, Didier Gourier

**Affiliations:** 1 Biogéochimie et Ecologie des Milieux Continentaux, UMR CNRS 7618, Université Pierre et Marie Curie, Paris, France; 2 Laboratoire de Chimie de la Matière Condensée de Paris, Ecole Nationale Supérieure de Chimie de Paris, UMR CNRS 7574, Paris, France

**Keywords:** Electron paramagnetic resonance, Lineshape, Solid state chemistry, Carbonaceous matter, Exobiology, Literate programming, Python

## Abstract

**Background:**

Electron Paramagnetic Resonance (EPR) is a non-destructive, non-invasive technique useful for the characterization of organic moieties in primitive carbonaceous matter related to the origin of life. The classical EPR parameters are the peak-to-peak amplitude, the linewidth and the *g* factor; however, such parameters turn out not to suffice to fully determine a single EPR line.

**Results:**

In this paper, we give the definition and practical implementation of a new EPR parameter based on the signal shape that we call the *R*_10_ factor. This parameter was originally defined in the case of a single symmetric EPR line and used as a new datation method for organic matter in the field of exobiology.

**Conclusion:**

Combined to classical EPR parameters, the proposed shape parameter provides a full description of an EPR spectrum and opens the way to novel applications like datation. Such a parameter is a powerful tool for future EPR studies, not only of carbonaceous matter, but also of any substance which spectrum exhibits a single symmetric line.

**Reproducibility:**

The paper is a literate program—written using Noweb within the Org-mode as provided by the Emacs editor— and it also describes the full data analysis pipeline that computes the *R*_10_ on a real EPR spectrum.

## Background: Necessity for a shape factor definition

In the field of exobiology, we need to determine the age of organic material in rock samples. Isotopic methods are commonly used to date the rock itself, but the organic matter may not be syngenetic with the rock. A novel solution based on Electron Paramagnetic Resonance (EPR) was proposed [1]; it requires the determination of a new EPR parameter, the *R*_10_, from the EPR spectrum of the rock sample, from which the age can be computed from an empirical log-linear correlation that was uncovered in [1]. Knowing the distribution of the different parameters that contribute to the *R*_10_, we may also provide a confidence interval for the age thus determined. In the following, we shall explain what the classical EPR parameters are and what the proposed new parameter brings to the table, and then describe the algorithm for the determination of the *R*_10_: how to process the data files generated during an EPR experiment, extract the classical EPR parameters and compute their distribution in order to have an estimate of their error; compute the new *R*_10_ parameter and its distribution from the preceding distributions. Thanks to this paper, scientists may themselves extract the *R*_10_ parameter from EPR data and use it not only for datation purposes but also to uniquely characterize the observed EPR spectrum lineshapes. Our goal is to automate a manual process that has proved scientifically successful yet cumbersome and tedious when applied on datasets that are getting larger. In that version of our code, some of our algorithmic choices just mirror the —successful— manual process. We have chosen the Python language because of its high level, ease of development and popularity; last but not least, it also provides powerful libraries for scientific development, and speed of execution turned out not to be a key factor for our goals^a^. The Python code runs inside the Sage computing platform [2], which aims at providing a single computing environment both for numerical and symbolic computations.

**Figure 1:**
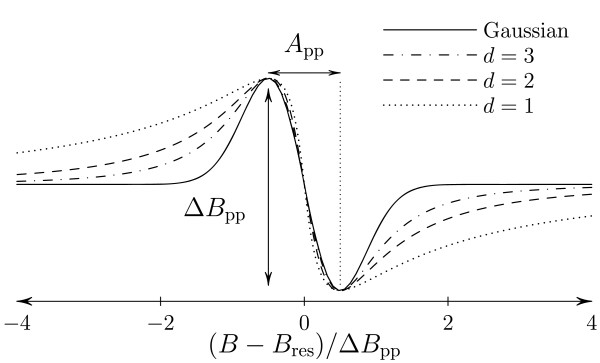
**Theoretical EPR lines corresponding to upper limit cases of dipolar broadening.** Continuous line: high spin concentration regime (Gaussian lineshape); Mixed line: diluted spin regime and 3D distribution (Lorentzian lineshape); Dashed line: diluted regime, 2D distribution (stretched Lorentzian); Dotted line: diluted regime, 1D distribution (stretched Lorentzian).

Electron Paramagnetic Resonance (EPR) is a non-destructive and non-invasive technique which has indeed long been used for the study of paramagnetic defects (organic radicals) in carbonaceous materials. Such defects have been detected with high sensitivity in coals by pioneering EPR works [3]. These types of radicals were therefore used for the characterization of a wide range of carbonaceous objects, ranging from coals [4-6] to cherts [7] through meteorites [8-11]. The EPR signal of kerogen is a single line, due to the presence of aromatic radical moieties, with an unpaired electron spin delocalized in carbon p-type molecular orbitals [4,9,12,13]. Several parameters can be deduced from an EPR spectrum, based on the amplitude *A*_pp_, the linewidth *ΔB*_pp_ and the resonance field *B*_res_ of the signal. However, for a single set of those three parameters, various lineshapes are possible (Figure 1); therefore, to fully determine the EPR line, a new EPR parameter, based on the lineshape, had to be defined.

The shape of the magnetic resonance absorption line of a system of interacting and randomly distributed spins depends on the nature of the interactions (dipole-dipole or exchange), on the spin concentration and on the dimensionality of the spatial distribution of the spins [14-18]. This study is restricted to the case of a dipole-dipole type interaction between electron spins, thus excluding exchange interaction occurring in very concentrated electron spin systems. Several limiting cases are distinguished in the literature, depending on the spin concentration and on the dimensionality of the distribution, cf. Table 1.

**Table 1:** **EPR lineshapes and lineshape parameter*R***_**10**_** for different limit regimes of dipolar broadening**

**Spin concentration**	**Distribution**	**Lineshape**	***R***_**10**_
High: dipolar and	3D	Gaussian to Lorentzian	≥0
hyperfine broadening			
Low: Lorentzian	0		
	2D	Stretched Lorentzian	−1.78
	1D	Stretched Lorentzian	−2.95

In the high concentration regime (generally considered when the fractional site occupation *r* by a paramagnetic centre exceeds 0.1), the lineshape is approximately Gaussian [17]. This regime also occurs when the line is broadened by unresolved hyperfine interaction. Given that EPR experimental spectra correspond to absorption derivatives, the Gaussian EPR line is described by:

(1) $$F_{G}(B-B_{\text{res}})=-A_{\text{pp}}\frac{B\;-\;B_{\text{res}}}{\Delta B_{\text{pp}}}\exp\left[-2{\left(\frac{B\;-\;B_{\text{res}}}{\Delta B_{\text{pp}}}\right)}^{2}\;+\;\frac{1}{2}\right]$$

where *B* is the applied magnetic field, *B*_res_ the field at the centre of the line (maximum of absorption), *A*_pp_ the peak-to-peak amplitude and *ΔB*_pp_ the peak-to-peak linewidth (Figure 1).

In the low concentration regime (generally considered when *r*<0.01) with no hyperfine broadening, the lineshape depends on the dimensionality of the spatial distribution of the paramagnetic centres [16]. When the distribution is random, the resonance line may be calculated from the relaxation function:

(2) $$G(d,t)=B\exp\left(-a\cdot t^{\frac{d}{3}}\right)$$

This function describes the decay with time *t* of the spin magnetization, perpendicular to the magnetic field, after an infinitely short microwave pulse. Parameter *a* is a constant that depends linearly on the spin concentration and parameter *d* represents the dimensionality of the spin distribution: *d*=1 for a linear distribution, *d*=2 for a distribution in a plane and *d*=3 for a distribution in a volume. The EPR absorption is the Fourier transform of the relaxation function, and thus the EPR spectrum is the field derivative of this Fourier transform:

(3) $$\begin{array}{l} F_{d}(B-B_{\text{res}})=ℜ\left[\overset{+\infty }{\underset{0}{\int }}G(d,t)\left(-\text{it}\frac{\mathrm{g\beta}}{\hbar }\right)\right)\\ \;\times \left(\exp\left[-i(B-B_{\text{res}})t\frac{\mathrm{g\beta}}{\hbar }\right]\text{dt}\right] \end{array}$$

where ℜ stands for the real part. In the case of a three dimensional distribution (*d*=3), the EPR lineshape function can be analytically calculated and corresponds to the field derivative of a Lorentzian function:

(4) $$\begin{array}{cc} F_{3}(B-B_{\text{res}})= & -\frac{16}{9}A_{\text{pp}}\frac{\frac{\left(B-B_{\text{res}}\right)}{\Delta B_{\text{pp}}}}{\left[\;1+\frac{4}{3}{\left(\frac{\left(B-B_{\text{res}}\right)}{\Delta B_{\text{pp}}}\right)}^{2}\right]} \end{array}$$

**Figure 2:**
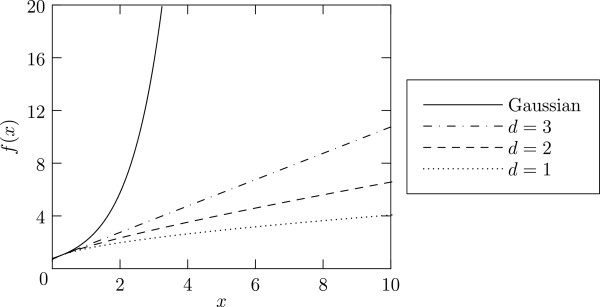
**Representation of the EPR spectra in the new *(x***_***Benc***_***,y***_***Benc***_***)* coordinates system described by **[14]** and given in equation**(7). Continuous line: Gaussian; mixed line: 3D distribution (Lorentzian); dashed line: 2D distribution (stretched Lorentzian); dotted line: 1D distribution (stretched Lorentzian).

For lower dimension of spin spatial distribution (*d*<3), the Fourier transform can only be calculated numerically. Figure 1 shows the theoretical EPR spectra corresponding to the Gaussian, Lorentzian (*d*=3) and low dimensional (*d*=1 and 2) cases. The wings of a Gaussian line fall off faster than those of a Lorentzian line while the wings of an EPR spectrum corresponding to a low-dimensional distribution fall off more slowly, giving rise to a so-called stretched Lorentzian lineshape. Originally, the *R*_10_ lineshape factor was imagined after studying the spectra in a coordinate system (*x*,*y*) in which the difference between the lineshapes stands out more clearly [14], and where the Lorentzian becomes a straight line:

(5) $$f_{L}(x)=x+\frac{3}{4}$$

and the Gaussian shape by an increasing exponential:

(6) $$f_{G}(x)=\exp(x-\frac{1}{4})$$

with *f*_G_(*x*)≥*f*_L_(*x*), ∀*x*, cf. Figure 2. That coordinate system can be obtained thanks to the following transformations as given in [14]:

(7) $$\begin{array}{l} x_{\text{Benc}}={\left(\frac{B-B_{\text{res}}}{\Delta B_{\text{pp}}}\right)}^{2}\text{and}\\ y_{\text{Benc}}=f(x_{\text{Benc}})=\sqrt{\frac{A_{\text{pp}}}{\left|F(B-B_{\text{res}})\right|}\frac{\left|B-B_{\text{res}}\right|}{\Delta B_{\text{pp}}}} \end{array}$$

where *F*=*F*_G_ or *F*_*d*_. We shall thus define two functions, one that creates the new abscissas from the old x ≡*B* and the other that creates the new ordinates from the old x and y ≡*F*(*B*−*B*_res_): 

Following the Noweb literate programming style as described in [19], the above code is called a *code chunk*, with a unique name given between angle brackets and followed with an equal sign, together with a corresponding unique number made up of the page number and a letter starting at *a* and increasing alphabetically on a given page; that number is mirrored in the left margin for easy reference. The number on the end of line after the code chunk name indicates the code chunk where the current code chunk is used. Often, we shall add some code to an already existing code chunk, and that will appear in two different ways: first, the name between angle brackets will be followed by an equal sign attached to a plus sign (instead of a lone equal sign), and the numbers on the end of line will also indicate where the code chunk gets some new code (a small triangle is added to that number, i.e. *⊲* for previous existing definition, and *⊳* for the next new code).

For diluted spin systems with low-dimensional distribution, the representative function *f* lies below the line corresponding to a Lorentzian shape. To quantitatively characterize the lineshape for systems intermediate between the above four ideal cases [Gaussian, Lorentzian (*d*=3), one-dimensional (*d*=1) and two-dimensional (*d*=2)], we define a lineshape parameter measuring the deviation from a Lorentzian line as described in [7]:

(8) $$R_{10}=\frac{1}{10}\int_{x=0}^{x=10}\left[\;f(x)-f_{L}(x)\right]\text{dx}$$

This parameter corresponds to the algebraic surface between the curve *f* representing an experimental EPR spectrum and the curve *f*_L_representing a Lorentzian line. *R*_10_is negative for a low-dimensional distribution (*d* < 3) and positive for an EPR line intermediate between Lorentzian and Gaussian lines (Table 1). The integration in equation (8) must be restricted to a finite range of x-values for the integral may not converge when *x*→*∞*. In practice, the range is limited to *x*≤10, since in most cases encountered the signal-to-noise ratio of the EPR spectra is poor for *x*≥10, inducing strong fluctuations in *f* and consequently in the lineshape parameter. Also, because of spectra with left/right assymmetry, the final *R*_10_is the average of the values computed on the left and right of the resonance field, i.e.

(9) $$R_{10}=\frac{1}{2}\left(R_{10}+R_{-10}\right)$$

To compute the integral in equation (8), we shall follow the method originally used: a simple top-left corner rectangular approximation. That allows full reproducibility with the original manual method that was used before automation with a program; in the future we may replace it with a more accurate algorithm if there is a general agreement on the need to depart from the manual processing. We shall thus consider a matrix matrixXYL —a numpy array— made up of the abscissas of the spectrum in the first column, the ordinates of the spectrum in the second column, and the ordinates of the ideal Lorentzian in the third column, with the number of lines corresponding to the number of data-points on the curves: 

The matrixXYL will be defined as a numpy array, and we use the sum function from the same library: 

In order to construct the matrix matrixXYL, we need the data abscissas and ordinates and we use equation 5 for the yL coordinates of the ideal Lorentzian curve: 

Again, we need to use the array data-structure, so we import it: 

Operationally, the *R*_10_was only defined separately for the parts of the curve which abscissas x are larger or smaller than the resonance field Bres, and we thus define an operator testSameSideofBres that will enable us to build two matrices matrixXYL, one for each side: 

In the case of the left hand side, we look for x lower than Bres, and the opposite for the right hand side: 

We shall thus obtain two values of *R*_10_, one for each side of *B*_res_, 

and we shall then use their average as the final value for the spectrum under study, cf. equation (9): 

We need to be careful with the order of the values in the matrix giving the coordinates in the new coordinate system defined in equation (7): if we start from small values of *x* in the original frame, then, for the left hand side of *B*_res_, values in the new frame will decrease, whereas values on the right hand side will increase. Thus, values on the left side must be reversed, whereas that will not be necessary for the right hand side. 

## Methods

All the relevant discussion about the experimental part of the work, that involves collecting EPR data on the rock samples, can be found in [1]. In the current paper, we focus on the specific data handling and processing in order to extract the *R*_10_parameter from an EPR spectrum and estimate the associated error. All computations were made in the Sage computing environment [2], with imports from the Numeric Python library [20].

In the spirit of reproducible research [21], the paper is written in the literate programming style [22]: the code and its explanation^b^are intertwined in a single place, and a particular program is then used to extract either the source code for execution on a computer or the literate paper for reading by humans. Literate programming tools exist, and we use Noweb [19] and Org-mode [23,24] within Emacs with Evil mode to enable vi commands. We also make use of the Sagetex package that comes with the Sage distribution, that allows Sage code to be executed when compiling the LaTeX source of the paper^c^, and we have a home-built script that manages to combine Org-mode with Sagetex together with a Noweb output. Figures are produced either with Sage and Sagetex, or with Asymptote: it allows us to program figures, and thus make them executable, and embeddable in the LaTeX source code. The code will be made available through the team’s website^d^.

## Processing data from an EPR file

### Removing the background signal

EPR spectra on which the *R*_10_factor was to be measured were selected for their symmetric and well-defined single absorption derivative signal. As usual in EPR studies, the large scale background signal was subtracted with a third degree polynomial fitted on the smooth parts of the spectrum where the signal variations are only due to noise, which in practice correspond to the first and last 10% data points in a typical spectrum. 

 From now on, the spectrum will be understood as the baseline corrected raw spectrum. 

### Reading the data for the spectra

EPR Spectra are given as .txt files, with a name made up of the following informations: 

•sample-name

•temperature-of-acquisition

•microwave-power

•number-of-scans

For example, gunflint_ambient_2mW_1scan.txt corresponds to a sample named gunflint, studied at ambient temperature with a microwave power of 2mW using 1 scan^e^. 

The first two lines must be skipped when loading data: they provide the EPR acquisition parameters and the file description. EPR text files comprise three columns, giving respectively the point index (starting from one and running to the total number of points recorded), the datapoint abscissa —the magnetic field *B*— and the datapoint ordinate —the intensity in arbitrary units. To ease data manipulation we extract two lists, abscissas and ordinates. 

and the load function loadtxt will be taken from the pylab library. 

**Figure 3:**
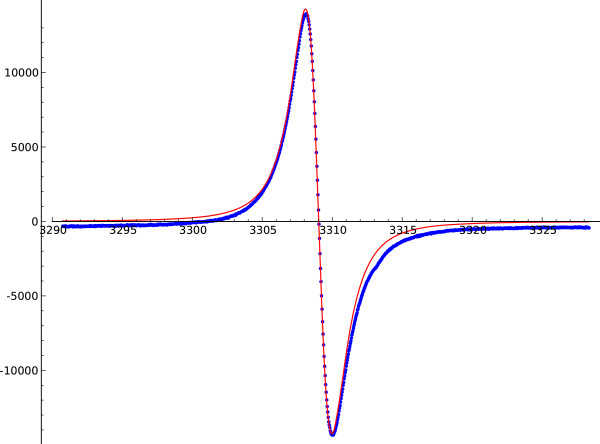
**The loaded EPR spectrum (dots) and the corresponding theoretical Lorentzian (continuous): the *R***_**10**_** factor is based on the integral difference between the two, cf. equation**(8).

We also have to make sure that the DATA variable is defined, which is normally automatic within Sage: 

In order to plot the spectrum as in Figure 3, we use Sage builtin plot function list_plot. 

## The distribution of the classical EPR parameters

To uncover the underlying Lorentzian curve which will be compared to the original spectrum for the *R*_10_computation, we need to find the three parameters that determine the latter: the peak-to-peak amplitude *A*_pp_, the linewidth *ΔB*_pp_and the resonance field *B*_res_. We define the peaks (positive and negative) as the extrema of the spectrum ordinate values, and the *A*_pp_and *ΔB*_pp_as the difference between the peaks’ ordinates and abscissas, respectively. 

The resonance field Bres was defined as the value at which the EPR lineshape crosses the baseline of the spectrum, which corresponds to the zero axis since the spectra are baseline corrected. 

The resonance field Bres is thus the mean of the two ordinates lying above and below the baseline respectively: 

Knowing the distributions of the classical EPR parameters App, DeltaBpp and Bres, we may check visually their normality thanks to a histogram plot; if normal, we may propagate their standard deviation in the global *R*_10_error calculation. 

In order to uncover the classical EPR parameters’ distributions, we chose the Monte Carlo error propagation method, cf. [25]: we take the measured spectrum, consider each data point as the mean of a random variable, then draw a new value for each data point given its distribution. For that, we suppose it is a normal distribution, with mean given by the data point and standard deviation given by the square root of the mean^f^; we thus use the normal distribution generator provided by randn in the pylab library. 

With this approach, a large number of cloned data sets is generated, for which App, DeltaBpp and Bres are computed; we then check for their normality by plotting their distribution and, if confirmed, compute their standard deviation for later use when computing the distribution of the *R*_10_. 

To store the parameters, we need to create the three empty lists listApp, listDeltaBpp and listBres. 

We then use the append function to add each calculated set of data to the storage lists.

For the Monte Carlo error propagation, we need to iterate a sufficient number of times in order to produce a significant set of data ; we thus create a global variable that specifies the number of Monte Carlo iterations. 

Because we add some noise during the Monte Carlo error propagation, and thus modify the original data, we need to store it before starting the Monte Carlo and retrieve it for each iteration in the Monte Carlo. 

## Extracting the new R_10_factor from the spectrum

The *R*_10_factor is calculated from the difference with the ideal Lorentzian derivative, which equation is:

(10) $$\frac{-A_{\text{pp}}\Delta B_{\text{pp}}^{3}(x-B_{\text{res}})}{{\left(\frac{3}{4}\Delta B_{\text{pp}}^{2}+{\left(x-B_{\text{res}}\right)}^{2}\right)}^{2}}$$

where *A*_pp_is the signal amplitude, *ΔB*_pp_the siglnal width and *B*_res_the resonance field; such an expression supposes that the background signal has been subtracted, i.e. that *A*_moy_=0. We thus compute the theoretical Lorentzian ordinates yL corresponding to the same abscissa as that of the spectrum and the same classical EPR parameters App, DeltaBpp and Bres as that of the spectrum; we store them in a list lorentzOrdinates. 

We plot the spectrum and its corresponding Lorentzian curve for visual checking. 

Now the *R*_10_parameter is computed relatively to the theoretical Lorentzian having the same set of classical EPR parameters, so we could compute the error on the former by propagating analytically the errors of the latter, which we now know thanks to the previous application of the Monte Carlo error propagation method. However, we found it easier and somewhat more in line with the computational approach to use a Monte Carlo approach to propagate the errors. We thus need to repeat the *R*_10_computation for a series of values of Bres, DeltaBpp and App to which we add a random error compatible with their distributions^g^: 

Because we modify the classical parameters during the *R*_10_ computation, we need to store the values and retrieve them before and after each iteration of the Monte Carlo: 

## Results and conclusion

We now have extracted the *R*_10_parameter together with its distribution and may proceed to use it, for example to determine the age of organic matter inside rock samples [1]. Given the distribution, we may then check if the mean and standard error do indeed properly characterize the parameter, and eventually assign a probability to a range of ages for the rock sample. The code runs in only a few minutes, if we take into account all the Monte Carlo computations. In [1], we demonstrate that the data processing as reported here can indeed provide us with a reasonable estimate for the age of rock samples older than 1 billion years.

## The complete code

## Endnotes

^a^Anyway, tools exist to go faster when needed, as Cython inside Sage that allows easy variable typing.

^b^Or maybe the explanation and its code… literate programming is really a whole new approach to writing, thinking and coding.

^c^This means that the outputs of some code need not be pasted inside the paper, but can be computed on the fly as needed.

^d^The url is http://hpu4science.org.

^e^This sample is part of the study where the *R*_10_parameter was proposed as a datation method [1].

^f^This corresponds to a normal distribution arising from a Poisson distribution, and is the common practice in EPR because of the underlying counting process when measuring the absorption giving the spectrum. We can indeed check it is so by studying the noise on the flat tails of EPR spectra.

^g^Using the Monte Carlo approach would also allow us to draw the values for the classical parameters according to their computed distribution.

## Competing interests

The authors declare that they have no competing interests.

## Authors’ contributions

MB carried out the experiments and did the analysis; MB and YLD developed the code and adapted the existing analysis framework to the new design, and wrote the paper. YLD initiated the move to a fully automated analysis framework for extracting the *R*_10_and proposed the Python language within the Sage environment. LB and DG designed the original *R*_10_parameter, corresponding manual extraction procedure and application to datation; they also took part in the analysis and helped with the experiments and the paper. All authors read and approved the final manuscript.
